# Public attitudes towards the implementation of pharmaceutical care services in community pharmacies – a 2025 nationwide cross-sectional survey among adults in Poland

**DOI:** 10.3389/fphar.2025.1706996

**Published:** 2025-10-08

**Authors:** Iwona Wrześniewska-Wal, Kuba Sękowski, Andrzej Silczuk, Mateusz Jankowski, Justyna Grudziąż-Sękowska

**Affiliations:** ^1^ School of Public Health, Centre of Postgraduate Medical Education, Warsaw, Poland; ^2^ Department of Community Psychiatry, Medical University of Warsaw, Warsaw, Poland

**Keywords:** pharmaceutical care, medication adherence, community pharmacy, chronic diseases, medical education, pharmacists

## Abstract

**Introduction:**

Pharmacists, as healthcare professionals, can improve treatment outcomes by providing pharmaceutical care services and implementing adherence-supporting tools. In Poland, these services are still at an early stage of development, and data on public attitudes toward them remain limited.

**Methods:**

A nationwide cross-sectional survey was conducted using the computer-assisted web interviews (CAWI) method on a stratified sample of 1,102 adults, representative in terms of gender, age, and place of residence. A validated questionnaire was used to assess attitudes toward pharmaceutical care services and adherence-supporting interventions. Sociodemographic and health-related factors were analyzed using multivariable logistic regression.

**Results:**

Attitudes toward pharmaceutical care were positive. The most frequently accepted services included drug interaction review (85.5%), explanation of dosing for new medications (83.5%), and monitoring therapy with previously unused medications (69.4%). Among adherence-supporting tools, instruction labels (85.5%) and simplified leaflets with pictograms (80.7%) were rated highest, followed by SMS or email reminders (59.2%) and additional pharmacist contact (46.7%–53.7%). Significant sociodemographic differences emerged: men more often valued shortened leaflets (83.1% vs. 77.8%), instruction labels (87.6% vs. 83.0%), and pharmacist follow-up (46.9% vs. 40.3%); respondents ≥60 years favored leaflets (85.2%) and labels (89.2%); parents more frequently accepted labels (87.8% vs. 81.6%). Higher education and better economic status were linked to more positive views of pharmaceutical care. Participants with chronic diseases more frequently accepted dosing explanations (86.1% vs. 80.4%), monitoring of new drugs (72.1% vs. 66.1%), and interaction reviews (88.1% vs. 82.2%), with diabetes or prediabetes emerging as the strongest predictor of positive attitudes. In multivariable analysis, male gender (aOR = 1.40; 95% CI = 1.10–1.79; p = 0.007) and good (aOR = 1.52; 95% CI = 1.04–2.21; p = 0.03) or average economic status (aOR = 1.64; 95% CI = 1.11–2.41; p = 0.01) were significantly associated with interest in free pharmacist consultations. Having children (aOR = 1.60; 95% CI = 1.15–2.23; p = 0.005) and occupational activity (aOR = 1.37; 95% CI = 1.03–1.81; p = 0.03) increased the likelihood of believing that a pharmacist consultation after receiving a prescription facilitates correct medication use.

**Conclusion:**

Implementing pharmacist consultations alongside simple adherence tools may significantly improve medication safety and treatment effectiveness in patients with chronic conditions.

## Background

Pharmaceutical care is a crucial element of the healthcare system, as pharmacotherapy is one of the most commonly used medical interventions. It is a systematic approach to medication use, aimed at improving patient health outcomes and enhancing quality of life (Hepler, 1996; Tácio de Mendonça Lima et al., 2018), and is defined as “the pharmacist’s contribution to patient care, aimed at optimising drug therapy and improving health outcomes” (Allemann et al., 2014).

Pharmaceutical care in community pharmacies takes different forms depending on the country. In the United States, its development has been ongoing for more than 50 years, with a major breakthrough marked by legislation that introduced prescription drug coverage and the financing of Medication Therapy Management (MTM) services (Urick and Meggs, 2019). MTM, provided by pharmacists, encompasses medication reviews, identification of drug interactions, patient education, and collaboration with physicians. In addition, community pharmacies in the United States offer a wide range of clinical services, including pharmacist consultations (Carter, 2016), vaccination and screening programs, prescribing hormonal contraception, and smoking cessation support (Salgado et al., 2020). In Canada (Raiche et al., 2020) and Australia (Laing et al., 2025; Tong et al., 2022), where the healthcare system is publicly funded, pharmacists actively participate in the care of patients with chronic diseases–diagnosing medication problems, issuing prescriptions, administering vaccinations, and providing health education.

The development of a patient-focused, rather than product-focused, model of care is also prevalent in many European countries. In the United Kingdom, within the centralized National Health Service (NHS) system, pharmacists identify medication-related issues, including interactions and treatment efficacy, and conduct comprehensive medication reviews, particularly in patients with multimorbidity and polypharmacy (Stewart et al., 2020; Crespo-Gonzalez et al., 2021). Pharmacies also offer health promotion services, such as smoking cessation programs, vaccinations, and contraceptive advice (Agomo et al., 2018). Similar solutions are being implemented in the Netherlands, Finland, and France, which, despite differences in financing mechanisms, strive to increase the role of pharmacists in patient care. This contributes to shortening waiting lists for primary care physicians and reducing the number of visits related to exacerbations of chronic diseases (Hedima and Okoro, 2023). However, regardless of differences in healthcare system financing mechanisms, many countries still experience a significant problem of non-adherence to treatment recommendations.

Pharmaceutical care plays an increasingly important role in the healthcare system, supporting patients in the proper use of prescribed therapies. Non-adherence to prescribed medication regimens in chronic diseases is a widespread problem. For example, between 43% and 65.5% of patients who fail to follow their prescribed treatment schedules are individuals with hypertension (Abegaz et al., 2017). The literature indicates that adherence rates vary substantially, ranging from 12.9% to 95.4% among patients with osteoporosis (Yeam et al., 2018). In other cases, a systematic review covering 27 studies demonstrated that adherence to pharmacological treatment in type 2 diabetes ranged from 38.5% to 93.1%, although only 22% of the studies reported adherence levels of ≥80% (Krass et al., 2015).

In its report “Adherence to Long-Term Therapies: Evidence for Action” (World Health Organization, 2003), the World Health Organization emphasizes that the lack of proper implementation of recommendations is one of the main factors limiting treatment effectiveness and generating additional healthcare costs (Cramer et al., 2008). In the same document, adherence is defined as the extent to which a patient’s medication use corresponds to the prescribed therapeutic regimen (World Health Organization, 2003). This concept encompasses a broader range of behavior, including medication intake, dietary adherence, and lifestyle modifications. Compliance, on the other hand, specifically refers to following the prescribed dose and timing of medications. Pharmaceutical care, through systematic medication reviews, counseling, and individualized support, plays a key role in improving both adherence and compliance, particularly among older patients and those undergoing polypharmacy (Cramer et al., 2008).

In Poland, pharmacists have competencies similar to those of their colleagues in the UK, France, and the Netherlands. Pursuant to the Pharmacist Profession Act (Journal of Laws of the Republic of Poland, 2021), pharmacists can provide healthcare services within the pharmaceutical care system, including conducting pharmaceutical consultations, reviewing medications, educating patients about drug interactions, performing diagnostic tests and measurements, qualifying and administering vaccinations recommended for adults, and issuing prescriptions. Importantly, Polish pharmacists are gaining additional qualifications through professional development courses. Between September 2023 and the first half of 2025, 2,041 pharmacists completed vaccination courses, and 2,364 pharmacists completed diagnostic testing courses (Wrześniewska-Wal, 2024; Centre of Postgraduate Medical Education and Training Coordination Office).

Despite the high competence of Polish pharmacists and the formal inclusion of pharmaceutical care in the Act as a health service, work is still underway to develop a financing model. In 2023, preparations were underway to implement the first pharmaceutical care service in Poland–a drug review. The service was piloted and assessed by the Agency for Health Technology Assessment and Tariff System, but the Agency’s Transparency Council issued a negative opinion (Transparency Council, 2023). Currently, pharmaceutical care is not financed by the public payer, the National Health Fund (NFZ), and remains a commercial service provided in pharmacies.

There is an ongoing public debate on how to introduce pharmaceutical care in Poland. Implementing it requires not only organizational and legislative changes but also an understanding of societal expectations regarding the role of pharmacists. Understanding how patients perceive pharmaceutical care and what their needs are can significantly help decision-makers develop an effective implementation strategy. Analyzing the factors determining willingness to use these services also allows us to identify the social groups most open to their use.

The aim of this study was therefore to characterize social attitudes towards the implementation of pharmaceutical care services in public pharmacies in Poland and to identify factors related to patients’ willingness to use these services.

## Materials and methods

### Study design

This nationwide cross-sectional survey was carried out using the computer-assisted web interviewing (CAWI) method. The study questionnaire included questions on public attitudes towards the implementation of pharmaceutical care services in community pharmacies. Data were collected by a specialized public opinion research company–Nationwide Research Panel Ariadna, Warsaw, Poland (Nationwide Research Panel Ariadna, 2025) – on behalf of the authors who provided the scientific background for this study. This study represents part of ongoing research on the implementation of pharmaceutical care in Poland, conducted by the School of Public Health, Centre for Medical Postgraduate Education, Warsaw, Poland.

Participants were recruited from a large, pre-verified online panel comprising more than 100,000 individuals. Sampling followed a stratified quota-based approach, with stratification parameters including sex, age, and urbanization level of place of residence (Groves et al., 2009). These parameters were based on current national demographic distributions as reported by Statistics Poland. The overall response rate was estimated at 22%. To maintain sampling integrity, if a respondent declined participation, another panel member matching the same stratification profile was invited in their place. Respondents were obligated to answer all the questions, so there were no missing answers.

This sampling strategy is consistent with that employed in prior population-level health and behavioral studies conducted in Poland, allowing for methodological comparability across different survey waves (Jankowski et al., 2023; Wrześniewska-Wal et al., 2024; Lewandowska et al., 2025). The sample size was calculated based on the demographic structure of the adult population of Poland and the number of participants was calculated as at least 1,000 individuals. A final sample of 1,102 adults was achieved.

The study protocol received approval from the Ethics Committee of the Centre of Postgraduate Medical Education (decision number 71/2025, dated 16 July 2025). The research adhered to the principles outlined in the Helsinki Declaration. Participation in this study was voluntary and anonymous, and informed consent was obtained from all participants. All procedures complied with the regulations specified in the Declaration of Helsinki.

### Measures

Public attitudes towards pharmaceutical care services were assessed using a self-prepared questionnaire based on a comprehensive literature review. An expert group reviewed the questionnaire for content validity and clarity. A pilot study was conducted with 8 adult participants who completed the questionnaire twice, 5 days apart, to assess test-retest reliability. Following the pilot study, two questions were revised to address the study objectives better and reduce potential response bias.

The questionnaire assessed multiple dimensions of pharmaceutical care attitudes:

− Current medication information practices;
− Perceived value of pharmacist consultation;
− Interest in free pharmacist consultation;
− Medication adherence support preferences, such as: shortened medication leaflet with colour-coded instructions; label-sticker on medication package with usage instructions; follow-up meeting with pharmacist 1–2 days after starting medication; text message (SMS) or email reminders about dosage and timing; phone or online contact with a pharmacist, and additional visit to the physician
−Interest in specific pharmaceutical care services, such as: explanation of dosage for unfamiliar medications; monitoring effects of new medications, and medication review to check for interactions or harmful combinations.

All attitude and preference questions used a five-point Likert scale, with consistent response options (definitely not, rather not, rather yes, definitely yes, not sure/difficult to tell) to facilitate comparison across items. For analytical purposes, responses were often dichotomised into positive (“rather yes” or “definitely yes”) *versus* other responses (Streiner et al., 2015).

The questionnaire items were presented with randomised rotation where applicable to minimise order effects and response bias.

### Statistical analysis

All statistical analyses were performed using IBM SPSS Statistics version 29 (Armonk, NY, United States). Descriptive statistics, including frequencies and percentages, were used to present the distribution of categorical variables. Comparisons between categorical variables were conducted using contingency tables and chi-square tests. Associations between 10 different variables and help-seeking behaviour in the event of mental disorders among adults in Poland were analysed using multivariable logistic regression analyses (Hosmer et al., 2013). Each variable was first assessed independently using bivariable logistic regression. Variables that demonstrated statistical significance in these bivariable analyses were subsequently entered into multivariable logistic regression models. Associations were expressed as odds ratios (OR) with corresponding 95% confidence intervals (95% CI). A p-value of less than 0.05 was considered indicative of statistical significance.

## Results

A total of 1,102 adults were included in the study, of whom 54.7% were women and 45.3% were men. The age group most frequently represented was 60 years or older (29.4%), while nearly half of the respondents had completed higher education (48.2%). Over half of the respondents reported being currently married (51.1%) and having children (62.0%). Most participants were occupationally active (63.5%) and rated their household economic status as good (48.6%) or moderate (38.0%). More than half (55.6%) reported at least one chronic disease. Detailed distributions of sociodemographic and clinical characteristics were presented in Table 1.

**TABLE 1:** Characteristics of the study population (N = 1,102).

Variable	n	%
Gender		
female	603	54.7
male	499	45.3
Age group [years]		
18–29	158	14.3
30–39	218	19.8
40–49	206	18.7
50–59	196	17.8
60+	324	29.4
Educational level		
primary	17	1.5
vocational	95	8.6
secondary	459	41.7
higher	531	48.2
Currently married		
yes	563	51.1
no	539	48.9
Place of residence		
rural area	413	37.5
city below 20,000 residents	145	13.2
city from 20,000 to 99,999 residents	220	20.0
city from 100,000 to 499,999 residents	184	16.7
city ≥500,000 residents	140	12.7
Having children		
yes	683	62.0
no	419	38.0
Occupational status		
active	700	63.5
passive	402	36.5
Self-reported household economic status		
good	536	48.6
moderate	419	38.0
bad	147	13.3
Having chronic diseases		
yes	613	55.6
no	489	44.4
Having at least one cardiovascular disease		
yes	319	28.9
no	783	71.1
Having diabetes or prediabetes		
yes	142	12.9
no	960	87.1
Having lung diseases (asthma or COPD)		
yes	68	6.2
no	1,034	93.8
Having at least one thyroid disease		
yes	165	15.0
no	937	85.0
Taking medications on a regular basis		
yes	503	45.6
no	599	54.4

Statistically significant values are bolded.

The majority of the studies’ participants reported positive attitudes towards pharmaceutical care services in community pharmacies. More than one-third of respondents (34.3%) reported that they “always” receive clear information from their doctor regarding new medications, whereas nearly one-quarter (23.5%) selected “often.” However, a notable percentage indicated suboptimal information: 18.5% “rarely” and 6.7% “never” received such instruction. Almost three-quarters of participants (73.5%) believed that a consultation with a pharmacist after receiving a prescription for an unfamiliar medication could help ensure correct use (“rather yes” or “definitely yes”: 73.5%). Interest in a free pharmacist consultation regarding medications, especially new ones, was expressed by just over half of respondents (“rather yes” or “definitely yes”: 51.4%). Regarding interventions to support medication adherence, the options most frequently endorsed as potentially helpful (“rather yes” or “definitely yes”) were: a label-sticker with usage instructions (85.5%) and a shortened, colour-coded leaflet (80.7%). Digital reminders (SMS/email) were seen as beneficial by 59.2%, while interest in follow-up contact with a pharmacist (e.g., meeting, phone, or online contact) ranged from 46.7% to 53.7%. Respondents also exhibited high interest in specific pharmaceutical care services: explanations of dosage for unfamiliar medicines (83.5%), monitoring medication effects (69.4%), and drug interaction review (85.5%). A detailed breakdown of respondents’ attitudes was presented in Table 2.

**TABLE 2:** Public attitudes towards the implementation of pharmaceutical care services in community pharmacies in Poland (N = 1,102).

Variable	n	%
When your doctor prescribes a medication that you have not taken before, do you receive clear information on how to use it (e.g., dosage, timing, interactions with other medications or food)?		
never	74	6.7
rarely	204	18.5
often	259	23.5
very often	187	17.0
always	378	34.3
Do you think that a consultation with a pharmacist at the pharmacy after receiving a prescription for a medication you have not taken before could help you use the medication correctly?		
definitely not	31	2.8
rather not	74	6.7
rather yes	517	46.9
definitely yes	293	26.6
not sure/difficult to tell	187	17.0
Would you be interested in a free consultation (discussion) with a pharmacist regarding the medications you are taking, especially a new one that you have not used before?		
definitely not	84	7.6
rather not	227	20.6
rather yes	380	34.5
definitely yes	186	16.9
not sure/difficult to tell	225	20.4
Could the following actions help you adhere to the regimen of a newly prescribed medication?		
Shortened medication leaflet with color-coded instructions		
definitely not	30	2.7
rather not	101	9.2
rather yes	445	40.4
definitely yes	444	40.3
not sure/difficult to tell	82	7.4
Label-sticker on the medication package with usage instructions		
definitely not	26	2.4
rather not	65	5.9
rather yes	422	38.3
definitely yes	520	47.2
not sure/difficult to tell	69	6.3
Follow-up meeting with a pharmacist 1–2 days after starting the medication		
definitely not	125	11.3
rather not	355	32.2
rather yes	327	29.7
definitely yes	150	13.6
not sure/difficult to tell	145	13.2
Text message (SMS) or email reminders about dosage and timing		
definitely not	104	9.4
rather not	239	21.7
rather yes	372	33.8
definitely yes	280	25.4
not sure/difficult to tell	107	9.7
Phone or online contact with a pharmacist		
definitely not	107	9.7
rather not	278	25.2
rather yes	387	35.1
definitely yes	205	18.6
not sure/difficult to tell	125	11.3
An additional visit to the physician		
definitely not	92	8.3
rather not	295	26.8
rather yes	394	35.8
definitely yes	206	18.7
not sure/difficult to tell	115	10.4
Would you be interested in the following services provided by a pharmacist at the community pharmacy?		
Explanation of dosage for a medication you have not used before		
definitely not	25	2.3
rather not	74	6.7
rather yes	451	40.9
definitely yes	470	42.6
not sure/difficult to tell	82	7.4
Monitoring the effects of a medication you have not used before		
definitely not	51	4.6
rather not	136	12.3
rather yes	422	38.3
definitely yes	343	31.1
not sure/difficult to tell	150	13.6
Reviewing your medications to check for interactions or harmful combinations		
definitely not	29	2.6
rather not	54	4.9
rather yes	426	38.7
definitely yes	516	46.8
not sure/difficult to tell	77	7.0

Statistically significant values are bolded.

### Socio-economic differences in public perception of adherence-promoting interventions

Having diabetes or prediabetes was the socioeconomic factor most strongly associated with positive public perceptions of multiple adherence support interventions, demonstrating the highest number of statistically significant results across the studied actions (Table 3). For other socioeconomic and health categories, significant differences, when present, typically pertained to isolated interventions. Females were less likely than males to perceive a shortened medication leaflet with color-coded instructions (83.1% vs. 77.8%, p = 0.03), a label-sticker on the medication package with usage instructions (87.6% vs. 83.0%, p = 0.03), and a follow-up meeting with a pharmacist 1–2 days after starting the medication (40.3% vs. 46.9%, p = 0.03) as helpful in supporting medication adherence. Respondents aged 60 years and above were more likely than those in younger age categories to perceive a shortened medication leaflet with color-coded instructions (85.2% for 60+ group; p = 0.04) and label-sticker instructions (89.2% for 60+ group; p = 0.003) as beneficial for adherence. Respondents with primary education most frequently indicated label-sticker instructions as beneficial (58.8%, p < 0.001), with statistically significant differences across education categories. Respondents with children were more likely than those without children to indicate that label-sticker instructions would help maintain adherence (87.8% vs. 81.6%, p = 0.004). Respondents reporting good economic status were more likely to select a shortened medication leaflet (81.5% vs. 70.7%, p = 0.004), a label-sticker (87.7% vs. 80.3%, p = 0.06), and a follow-up pharmacist meeting (40.9% vs. 38.8%, p = 0.04) as beneficial compared to those with bad economic status. A higher proportion of respondents with active occupational status indicated that SMS/email reminders (62.6% vs. 53.2%, p = 0.002) and phone/online pharmacist contact (56.0% vs. 49.8%, p = 0.04) could support adherence compared to those passive occupationally. Respondents with chronic diseases were more likely to view SMS/email reminders (60.4% vs. 57.7%, p = 0.4), phone/online pharmacist contact (56.9% vs. 49.7%, p = 0.02), and an additional physician visit (58.6% vs. 49.3%, p = 0.002) as helpful. Among those with cardiovascular disease, the prevalence of perceiving shortened leaflet and label-sticker interventions as beneficial was higher, but significant only for the label-sticker (87.8% vs. 84.5%, p = 0.2) and follow-up pharmacist meeting (44.8% vs. 42.7%, p = 0.5). Respondents with diabetes or prediabetes were more likely than those without to select the shortened leaflet (89.4% vs. 79.4%, p = 0.005), label-sticker (93.0% vs. 84.4%, p = 0.007), follow-up meeting (51.4% vs. 42.1%, p = 0.04), SMS/email reminders (67.6% vs. 57.9%, p = 0.03), phone/online pharmacist contact (60.6% vs. 52.7%, p = 0.08), and additional physician visit (62.0% vs. 53.3%, p = 0.05) as beneficial. Thus, having diabetes or prediabetes was associated with the most statistically significant affirmative perceptions of possible adherence interventions. Only the additional physician visit was more frequently selected by those with lung diseases (67.6% vs. 53.6%, p = 0.02). There were no significant differences for other variables. There were no significant differences in the perception of actions supporting medication adherence by marital status, place of residence, thyroid disease status, or regular medication use. Additionally, no significant differences were observed for the other interventions assessed.

**TABLE 3:** Socio-economic differences in public perception of actions that may help people to adhere to the regimen of a newly prescribed medication (N = 1,102).

Could the following actions help you adhere to the regimen of a newly prescribed medication? – Positive answers („definitely yes” or „rather yes”)												
	Shortened medication leaflet with color-coded instructions		Label-sticker on the medication package with usage instructions		Follow-up meeting with a pharmacist 1–2 days after starting the medication		Text message (SMS) or email reminders about dosage and timing		Phone or online contact with a pharmacist		An additional visit to the physician	
Variable	%	p	%	p	%	p	%	p	%	p	%	p
Gender												
female (n = 603)	83.1	**0.03**	87.6	**0.03**	40.3	**0.03**	57.0	0.1	53.6	0.9	54.2	0.9
male (n = 499)	77.8		83.0		46.9		61.7		53.9		54.7	
Age group [years]												
18–29 (n = 158)	77.2	**0.04**	80.4	**0.003**	42.4	0.2	61.4	0.1	56.3	0.7	56.3	0.9
30–39 (n = 218)	77.5		79.8		49.5		61.0		55.0		56.0	
40–49 (n = 206)	76.7		85.0		45.6		62.6		52.9		54.9	
50–59 (n = 196)	83.7		90.3		40.3		61.7		56.1		54.1	
60+ (n = 324)	85.2		89.2		39.8		53.1		50.6		52.5	
Educational level												
primary (n = 17)	76.5	0.5	70.6	**<0.001**	58.8	0.5	82.4	0.3	64.7	0.5	76.5	0.3
vocational (n = 95)	74.7		80.0		45.3		56.8		48.4		55.8	
secondary (n = 459)	81.5		82.4		44.0		59.3		53.2		53.8	
higher (n = 531)	81.2		89.6		41.8		58.8		54.8		54.0	
Currently married												
yes (n = 563)	82.1	0.2	87.2	0.1	43.5	0.9	59.7	0.7	54.5	0.6	54.4	0.9
no (n = 539)	79.2		83.7		43.0		58.6		52.9		54.5	
Place of residence												
rural area (n = 413)	79.9	0.6	85.7	0.9	43.3	0.9	61.7	0.3	53.0	0.9	51.3	0.2
city below 20,000 residents (n = 145)	77.9		83.4		45.5		51.0		52.4		53.1	
city from 20,000 to 99,999 residents (n = 220)	83.6		87.3		44.5		60.5		55.5		57.7	
city from 100,000 to 499,999 residents (n = 184)	82.6		85.3		42.9		59.2		55.4		60.3	
city ≥500,000 residents (n = 140)	78.6		84.3		39.3		57.9		52.1		52.1	
Having children												
yes (n = 683)	81.3	0.5	87.8	**0.004**	44.1	0.5	60.6	0.2	55.3	0.2	55.5	0.4
no (n = 419)	79.7		81.6		42.0		56.8		51.1		52.7	
Occupational status												
active (n = 700)	80.3	0.7	84.4	0.2	44.7	0.2	62.6	**0.002**	56.0	**0.04**	55.4	0.4
passive (n = 402)	81.3		87.3		40.8		53.2		49.8		52.7	
Self-reported household economic status												
good (n = 536)	81.5	**0.004**	87.7	0.06	40.9	**0.04**	59.0	0.9	52.1	**0.02**	51.3	0.1
moderate (n = 419)	83.1		84.5		48.0		59.4		58.5		58.0	
bad (n = 147)	70.7		80.3		38.8		59.2		46.3		55.8	
Having chronic diseases												
yes (n = 613)	82.4	0.1	86.9	0.1	43.9	0.7	60.4	0.4	56.9	**0.02**	58.6	**0.002**
no (n = 489)	78.5		83.6		42.5		57.7		49.7		49.3	
Having at least one cardiovascular disease												
yes (n = 319)	84.0	0.07	87.8	0.2	44.8	0.5	58.9	0.9	54.9	0.6	57.4	0.2
no (n = 783)	79.3		84.5		42.7		59.3		53.3		53.3	
Having diabetes or prediabetes												
yes (n = 142)	89.4	**0.005**	93.0	**0.007**	51.4	**0.04**	67.6	**0.03**	60.6	0.08	62.0	0.05
no (n = 960)	79.4		84.4		42.1		57.9		52.7		53.3	
Having lung diseases (asthma or COPD)												
yes (n = 68)	83.8	0.5	85.3	0.9	50.0	0.2	61.8	0.7	55.9	0.7	67.6	**0.02**
no (n = 1,034)	80.5		85.5		42.8		59.0		53.6		53.6	
Having at least one thyroid disease												
yes (n = 165)	82.4	0.5	85.5	0.9	40.0	0.4	57.0	0.5	60.0	0.08	57.6	0.4
no (n = 937)	80.4		85.5		43.9		59.6		52.6		53.9	
Taking medications on a regular basis												
yes (n = 503)	82.5	0.2	87.1	0.2	42.1	0.5	58.4	0.7	55.9	0.2	56.7	0.2
no (n = 599)	79.1		84.1		44.2		59.8		51.9		52.6	

Statistically significant values are bolded.

### Socio-economic differences in public perception of selected pharmaceutical care services

The proportion of respondents interested in reviewing medications to check for interactions or harmful combinations was significantly higher among those aged 40–49 years (87.4%) compared to younger groups (p = 0.01) (Table 4). No other age-based differences reached statistical significance. Having chronic diseases and having diabetes or prediabetes yielded the most statistically significant associations with positive perceptions of all pharmaceutical care interventions. Respondents with chronic diseases demonstrated significantly greater interest in the explanation of dosage for a medication not used before (86.1% vs. 80.4%; p = 0.01), monitoring the effects of a medication not used before (72.1% vs. 66.1%; p = 0.03), and reviewing medications to check for interactions or harmful combinations (88.1% vs. 82.2%; p = 0.006). Those diagnosed with diabetes or prediabetes expressed significantly greater interest than others in all three services all those services (93.0% vs. 82.2%; p = 0.001; 80.3% vs. 67.8%; p = 0.03, and 92.3% vs. 84.5%; p = 0.01 respectively). Having at least one cardiovascular disease was positively associated with higher interest in explanation of dosage (87.5% vs. 82.0%; p = 0.03) and monitoring effects of a new medication (74.0% vs. 67.6%; p = 0.04). No statistically significant differences were found for other variables in relation to any of the assessed services (all p > 0.05).

**TABLE 4:** Socio-economic differences in public perception of selected pharmaceutical care services that may be provided by a pharmacist at the community pharmacy (N = 1,102).

Would you be interested in the following services provided by a pharmacist at the community pharmacy? – Positive answers („definitely yes” or „rather yes”)						
	Explanation of dosage for a medication you have not used before		Monitoring the effects of a medication you have not used before		Reviewing your medications to check for interactions or harmful combinations	
Variable	%	p	%	p	%	p
Gender						
female (n = 603)	82.9	0.5	67.7	0.2	86.9	0.1
male (n = 499)	84.4		71.5		83.8	
Age group [years]						
18–29 (n = 158)	82.3	0.3	67.1	0.7	84.8	**0.01**
30–39 (n = 218)	79.4		70.6		79.4	
40–49 (n = 206)	85.0		71.8		87.4	
50–59 (n = 196)	87.2		70.9		91.3	
60+ (n = 324)	84.0		67.3		85.2	
Educational level						
primary (n = 17)	76.5	0.2	64.7	0.9	76.5	0.6
vocational (n = 95)	76.8		72.6		83.2	
secondary (n = 459)	83.9		69.7		86.1	
higher (n = 531)	84.7		68.7		85.7	
Currently married						
yes (n = 563)	85.3	0.1	70.2	0.6	87.0	0.1
no (n = 539)	81.8		68.6		83.9	
Place of residence						
rural area (n = 413)	84.5	0.4	70.7	0.7	86.7	0.5
city below 20,000 residents (n = 145)	84.1		70.3		83.4	
city from 20,000 to 99,999 residents (n = 220)	83.6		70.5		84.1	
city from 100,000 to 499,999 residents (n = 184)	85.3		68.5		88.0	
city ≥500,000 residents (n = 140)	77.9		64.3		82.9	
Having children						
yes (n = 683)	84.8	0.2	71.0	0.1	86.7	0.2
no (n = 419)	81.6		66.8		83.5	
Occupational status						
active (n = 700)	83.6	0.9	71.3	0.08	85.7	0.8
passive (n = 402)	83.6		66.2		85.1	
Self-reported household economic status						
good (n = 536)	84.3	0.4	68.8	0.2	87.3	0.2
moderate (n = 419)	84.0		72.1		84.2	
bad (n = 147)	79.6		63.9		82.3	
Having chronic diseases						
yes (n = 613)	86.1	**0.01**	72.1	**0.03**	88.1	**0.006**
no (n = 489)	80.4		66.1		82.2	
Having at least one cardiovascular disease						
yes (n = 319)	87.5	**0.03**	74.0	**0.04**	88.4	0.08
no (n = 783)	82.0		67.6		84.3	
Having diabetes or prediabetes						
yes (n = 142)	93.0	**0.001**	80.3	**0.03**	92.3	**0.01**
no (n = 960)	82.2		67.8		84.5	
Having lung diseases (asthma or COPD)						
yes (n = 68)	83.8	0.9	70.6	0.8	89.7	0.3
no (n = 1,034)	83.6		69.3		85.2	
Having at least one thyroid disease						
yes (n = 165)	86.7	0.2	70.9	0.7	83.6	0.5
no (n = 937)	83.0		69.2		85.8	
Taking medications on a regular basis						
yes (n = 503)	84.9	0.3	71.6	0.2	87.3	0.1
no (n = 599)	82.5		67.6		84.0	

Statistically significant values are bolded.

In multivariable logistic regression analysis (Table 5), having children (aOR = 1.60; 95%CI = 1.15–2.23; p = 0.005), active occupational status (aOR = 1.37; 95%CI = 1.03–1.81; p = 0.03), and good self-reported household economic status (aOR = 1.92; 95%CI = 1.28–2.87; p = 0.002) were significantly associated with beliefs that consultation with a pharmacist at the pharmacy after receiving a prescription for a medication patients have not taken before could help them use the medication correctly.

**TABLE 5:** Factors associated with public attitudes towards consultation with a pharmacist at the pharmacy after receiving a prescription for a medication (N = 1,102).

Do you think that a consultation with a pharmacist at the pharmacy after receiving a prescription for a medication you have not taken before could help you use the medication correctly? – „rather yes” or „definitely yes”						
			Bivariable logistic Regression		Multivariable logistic Regression	
Variable	%	p	OR (95%CI)	p	aOR (95%CI)	p
Gender						
female (n = 603)	72.6	0.5	0.91 (0.69–1.19)	0.5		
male (n = 499)	74.5		Reference			
Age group [years]						
18–29 (n = 158)	70.3	0.3	0.79 (0.52–1.20)	0.3		
30–39 (n = 218)	69.3		0.75 (0.51–1.10)	0.1		
40–49 (n = 206)	77.7		1.16 (0.77–1.75)	0.5		
50–59 (n = 196)	74.0		0.95 (0.63–1.42)	0.8		
60+ (n = 324)	75.0		Reference			
Having higher education						
yes (n = 531)	72.3	0.4	0.90 (0.68–1.16)	0.4		
no (n = 571)	74.6		Reference			
Currently married						
yes (n = 563)	76.6	**0.02**	1.38 (1.05–1.80)	0.02	1.00 (0.72–1.39)	0.9
no (n = 539)	70.3		Reference		Reference	
Place of residence						
rural area (n = 413)	74.8	0.3	1.11 (0.72–1.71)	0.7		
city below 20,000 residents (n = 145)	75.9		1.17 (0.69–1.99)	0.6		
city from 20,000 to 99,999 residents (n = 220)	75.0		1.12 (0.69–1.81)	0.7		
city from 100,000 to 499,999 residents (n = 184)	67.4		0.77 (0.48–1.25)	0.3		
city ≥500,000 residents (n = 140)	72.9		Reference			
Having children						
yes (n = 683)	76.9	**0.001**	1.56 (1.19–2.05)	0.001	1.60 (1.15–2.23)	**0.005**
no (n = 419)	68.0		Reference		Reference	
Occupational status						
active (n = 700)	75.7	**0.03**	1.36 (1.03–1.79)	0.03	1.37 (1.03–1.81)	**0.03**
passive (n = 402)	69.7		Reference		Reference	
Self-reported household economic status						
good (n = 536)	77.8	**0.001**	2.04 (1.37–3.01)	<0.001	1.92 (1.28–2.87)	**0.002**
moderate (n = 419)	71.6		1.46 (0.98–2.18)	0.06	1.45 (0.97–2.16)	0.07
bad (n = 147)	63.3		Reference		Reference	
Having chronic diseases						
yes (n = 613)	74.6	0.4	1.13 (0.86–1.48)	0.4		
no (n = 489)	72.2		Reference			
Taking medications on a regular basis						
yes (n = 503)	75.0	0.3	1.15 (0.88–1.50)	0.3		
no (n = 599)	72.3		Reference			

Statistically significant values are bolded.

In multivariable logistic regression analysis (Table 6), male gender (aOR = 1.40; 95%CI = 1.10–1.79; p = 0.007), and good (aOR = 1.52; 95%CI = 1.04–2.21; p = 0.03) or moderate (aOR = 1.64; 95%CI = 1.11–2.41; p = 0.01) self-reported household economic status were significantly associated with public interest in a free consultation (discussion) with a pharmacist regarding the medications taken and pharmaceutical care.

**TABLE 6:** Factors associated with interest in a free consultation (discussion) with a pharmacist regarding the medications taken and pharmaceutical care (N = 1,102).

Would you be interested in a free consultation (discussion) with a pharmacist regarding the medications you are taking, especially a new one that you have not used before? – „rather yes” or „definitely yes”						
			Bivariable logistic Regression		Multivariable logistic Regression	
Variable	%	p	OR (95%CI)	p	aOR (95%CI)	p
Gender						
female (n = 603)	47.6	**0.006**	Reference	0.006	Reference	**0.007**
male (n = 499)	55.9		1.40 (1.10–1.77)		1.40 (1.10–1.79)	
Age group [years]						
18–29 (n = 158)	43.7	0.1	Reference		Reference	
30–39 (n = 218)	50.0		1.29 (0.85–1.95)	0.2	1.16 (0.76–1.77)	0.5
40–49 (n = 206)	57.3		1.73 (1.14–2.63)	0.01	1.45 (0.92–2.26)	0.1
50–59 (n = 196)	54.6		1.55 (1.02–2.36)	0.04	1.26 (0.79–2.00)	0.3
60+ (n = 324)	50.3		1.31 (0.89–1.91)	0.2	0.95 (0.61–1.49)	0.8
Having higher education						
yes (n = 531)	51.4	0.9	1.00 (0.79–1.27)	0.9		
no (n = 571)	51.3		Reference			
Currently married						
yes (n = 563)	53.6	0.1	1.21 (0.95–1.53)	0.1		
no (n = 539)	49.0		Reference			
Place of residence						
rural area (n = 413)	52.1	0.9	1.09 (0.74–1.59)	0.7		
city below 20,000 residents (n = 145)	52.4		1.10 (0.69–1.75)	0.7		
city from 20,000 to 99,999 residents (n = 220)	51.8		1.08 (0.70–1.64)	0.7		
city from 100,000 to 499,999 residents (n = 184)	49.5		0.98 (0.63–1.52)	0.9		
city ≥500,000 residents (n = 140)	50.0		Reference			
Having children						
yes (n = 683)	53.7	**0.04**	1.28 (1.01–1.64)	0.04	1.24 (0.93–1.65)	0.1
no (n = 419)	47.5		Reference		Reference	
Occupational status						
active (n = 700)	53.6	0.05	1.28 (0.99–1.63)	0.05		
passive (n = 402)	47.5		Reference			
Self-reported household economic status						
good (n = 536)	52.1	0.08	1.45 (1.01–2.09)	0.04	1.52 (1.04–2.21)	**0.03**
moderate (n = 419)	53.5		1.53 (1.05–2.24)	0.03	1.64 (1.11–2.41)	**0.01**
bad (n = 147)	42.9		Reference		Reference	
Having chronic diseases						
yes (n = 613)	55.3	**0.003**	1.43 (1.13–1.81)	0.003	1.36 (0.89–2.08)	0.1
no (n = 489)	46.4		Reference		Reference	
Taking medications on a regular basis						
yes (n = 503)	55.9	**0.006**	1.40 (1.10–1.77)	0.006	1.12 (0.73–1.72)	0.6
no (n = 599)	47.6		Reference		Reference	

Statistically significant values are bolded.

## Discussion

The study revealed positive public attitudes toward pharmaceutical care services provided in community pharmacies. Consultations related to the analysis of potential drug interactions (85.5%), clarification of new medication dosing (83.5%), and monitoring the effects of a medication not previously used by the participant (69.4%) were the most popular. Simple tools supporting treatment adherence, such as labels and concise information leaflets, also received particularly high support. Importantly, demographic and health factors had a limited impact on attitudes toward pharmaceutical services, although men were more likely to express interest in a free consultation. The strongest predictor of a positive assessment of the proposed solutions was the presence of diabetes or prediabetes, confirming a greater willingness of individuals with chronic conditions to utilize integrated pharmacist support.

The study revealed significant gaps in providing patients with clear information on the use of newly prescribed medications, as 25% reported that such information is rarely or never provided. These findings are consistent with international observations, which indicate that the limited duration of medical visits constitutes a major barrier to effective communication about medications, with only about half of patients reporting full understanding of the recommendations. In Germany, the average consultation lasts 7.6 min, while in the United States it is 15.9 min (Deveugele et al., 2002) (Irving et al., 2017). In Poland, the public payer–the National Health Fund (NFZ) – does not formally define a minimum or average duration of family physician or specialist visits. According to a now-obsolete NFZ regulation from 2008, the average duration of a specialist consultation was assumed to be 15–20 min (Regulation No). However, medical documentation and administrative tasks consume nearly one-third of the consultation time in Poland (Supreme Audit Office NIK, 2021). International studies consistently demonstrate that short consultations contribute to polypharmacy, overuse of antibiotics, and insufficient patient communication (Irving et al., 2017). Lack of understanding of medical recommendations may lead to dosing errors and reduced adherence, as confirmed by numerous studies–for example, among patients with diabetes and low health literacy (Schillinger et al., 2003), as well as among parents presenting to emergency departments due to dosing errors with liquid medications (Yin et al., 2014).

The study demonstrated that respondents positively evaluated simple forms of support facilitating proper medication administration. Over 80% of participants highly rated shortened, colour-coded leaflets and package labels containing specific instructions. Similar findings were reported in primary care settings in Chicago, Illinois, and Shreveport, Louisiana (Wolf et al., 2010), where studies confirmed that simplified educational materials with graphic elements significantly improved adherence to medication. The present study also showed that respondents with children were more likely than those without children to indicate that clear labels and pictogram-enhanced instructions would support adherence (p = 0.004). At King Saud University Medical City in Riyadh (Algabbani et al., 2022), caregivers of children found the addition of visual aids to leaflets helpful in understanding medication instructions. Importantly, caregivers who received combined text-and-pictogram instructions made significantly fewer errors than those who received text-only instructions (Yin et al., 2011).

The study found that individuals with a favorable economic situation were more likely to report benefits related to medication adherence through the use of additional support tools, such as a shortened leaflet accompanying the medication (p = 0.004) and a label and sticker (p = 0.06), compared to those with a lower economic situation. These results are consistent with observations from international studies. In the case of antidiabetic medications, five studies conducted in Asia and four studies from the United States confirmed a positive association between higher income and medication adherence. However, there are also exceptions–a single Malaysian study found an inverse relationship, while fifteen studies from various regions found no significant association between the economic situation and medication adherence (Studer et al., 2023). The present study shows that the group of patients who declared a high economic status was also more likely to rate their subsequent visit to a pharmacist as favorable.

In this study, respondents highlighted digital medication reminders such as text messages and emails. Although these are among the oldest forms of e-health interventions and are less effective than newer, interactive tools (e.g., mobile apps, telemonitoring platforms, or wearable devices), they still provide important support for proper medication adherence, especially in resource-limited countries (LMICs) (Pouls et al., 2021). For example, high acceptance of such solutions was reported in Tanzania (Edward et al., 2021) and Ethiopia (Zewdu et al., 2024), where as many as 64.5% of patients with hypertension declared willingness to use text message reminders. The results of this study indicate a similar trend, with approximately 60% of respondents expressing approval for SMS reminders or telephone contact, which is consistent with the findings in Ethiopia. However, what is new in this study is that respondents who preferred SMS/email reminders also expressed interest in more direct and in-depth contact with a pharmacist (e.g., a meeting, a phone call, or online contact), with 46.7%–53.7% of participants indicating such a preference.

In an Indonesian study, consultations were rated as the most highly valued pharmaceutical service for both patients and pharmacists, with the aim of improving adherence among patients with diabetes. Other services, such as brochures, medication reviews, and group discussions with patients, can also be provided in conjunction with consultations (Presley et al., 2021). The present study shows that nearly 70% of respondents believed that a consultation with a pharmacist after receiving a prescription for a new medication could facilitate proper adherence to therapy. Meta-analyses indicate that patients prefer clear, detailed, and easily accessible information about adverse drug reactions (ADRs), as adherence decisions are made based on the balance of risks and benefits (EL Masri et al., 2022).

The factors influencing patients’ decisions vary more according to disease progression and prior treatment history than to the time since diagnosis (EL Masri et al., 2022). Knowledge of medications also played a significant role (Sieling et al., 2025). Importantly, willingness to utilize pharmaceutical care in this context increases markedly when the service is personalized and addresses a specific health concern (Bundogji et al., 2022).

Nearly all pharmacist interventions that effectively improved adherence—for example, in the case of antihypertensive drugs—were complex and included a combination of different strategies (Morgado et al., 2011; Stewart et al., 2014). The results of this study confirm this trend: respondents expressed particular interest in pharmaceutical care services, especially drug interaction analysis (85.5%), explanations of dosages of newly prescribed drugs (83.5%), and monitoring of their effects (69.4%).

Researchers in Canada and Nigeria observed that information about the proper use of medicines is more effective than information about side effects, as discussing side effects may have the opposite effect, negatively influencing patients’ attitudes and reducing adherence (Eze et al., 2023). This means that not only the pharmacist consultation itself, but also the way pharmacists communicate these benefits and risks, can affect patients’ decisions about adherence (Dyck et al., 2005).

Furthermore, patients’ willingness to use pharmaceutical care services may depend on demographic and socio-economic factors. Polish studies on trust in pharmacists have shown that individuals reporting a good economic situation were significantly more likely to trust pharmacists and to choose a pharmacy as a place of healthcare provision (Wrześniewska-Wal et al., 2025). The influence of demographic characteristics on willingness to use such services was also confirmed in an analysis of data from Malaysia: women, individuals with higher education, urban residents, and those with self-reported health problems were more likely to use pharmacy services (Hamidi et al., 2021). Similarly, tailoring services to individual patient needs–for example, pharmacist consultations during the initiation of a new medication–was shown to depend on education level in a study conducted in South Korea (Kang et al., 2017). In the present study, factors such as having children, active employment status, and good economic situation were significantly associated with the choice of a pharmacist consultation after receiving a new prescription from a physician (p < 0.05). However, in multivariate analysis, some of these effects were attenuated. The lack of significant differences related to sex or education suggests that the need for this type of support is relatively evenly distributed across the population. Interest in participating in a free pharmacist consultation was approximately 50%, with men being significantly more likely to express willingness to participate (p = 0.007).

In this study, there were no questions on pharmaceutical care services that involved co-payment by the patient. However, in many European countries, patients pay for certain pharmaceutical care services out of their own pockets (Tácio de Mendonça Lima et al., 2018; Salgado et al., 2020; Raiche et al., 2020; Laing et al., 2025; Tong et al., 2022; Stewart et al., 2020). In Poland, there is no possibility of co-payment for health services funded within the mandatory health insurance. The introduction of co-payment by the patient for pharmaceutical care services will require dedicated law regulation and investigation in the further studies.

### Practical implications

This study has several important practical implications for the development of pharmaceutical care in Poland. First, nationwide data on public attitudes toward pharmaceutical services can provide a basis for policymakers in implementing pharmacy-based programs aimed at improving patient safety and promoting adherence to therapeutic recommendations. Second, the high interest in consultations related to potential drug interactions, clarification of new medication dosing, and monitoring effects of new medications highlights key areas where pharmacists can actively contribute to patient care. Third, simple adherence-support tools, such as pictograms and concise information leaflets, may be effectively implemented to enhance understanding and proper medication use, especially among patients with chronic conditions like diabetes or prediabetes. Fourth, demographic and health characteristics had a limited impact on attitudes, suggesting that interventions to expand pharmacist-led services could be broadly accepted across different population groups. Finally, these findings support the need for legal and financial frameworks to incentivise and integrate pharmacists’ services within the national healthcare system.

### Limitations

This cross-sectional survey has some limitations. The data were self-reported, which may introduce minor inaccuracies in recall. Participation required Internet access, which is generally widespread in Poland, making this limitation less impactful. The study focused on selected pharmaceutical services and adherence-support tools, so findings may not fully represent all possible pharmacist-led interventions. Health conditions were self-reported and not cross-checked with medical records. CAWI method was used so those without Internet access were excluded from the study. Finally, while associations with sociodemographic and health characteristics were explored, causal relationships cannot be inferred due to the cross-sectional design.

## Conclusion

Based on the study results, pharmaceutical care services, such as reviewing drug interactions, explaining dosing of new medications, and monitoring therapy outcomes, are highly accepted by the public. Simple adherence-supporting tools, including instruction labels and simplified pictogram-based leaflets, were rated as particularly effective in improving treatment adherence. Significant sociodemographic differences were observed–older adults, individuals with children, and those with higher socioeconomic status more frequently indicated these interventions as helpful. The strongest predictor of positive perceptions of both pharmaceutical care services and adherence-supporting tools was the presence of chronic conditions, particularly diabetes and prediabetes. These findings suggest that implementing pharmacist consultations in combination with simple adherence tools can substantially improve pharmacotherapy safety and treatment effectiveness. The adoption of such solutions within the healthcare system could contribute to optimizing care for patients burdened with chronic diseases.

## Data Availability

The original contributions presented in the study are included in the article/supplementary material, further inquiries can be directed to the corresponding author.
